# Association Between Lipid Biomarkers, Physical Activity, and Socioeconomic Status in a Population-Based Cross-Sectional Study in the UK

**DOI:** 10.1186/s40798-016-0049-9

**Published:** 2016-06-13

**Authors:** Heather Brown, Frauke Becker, Kofi Antwi

**Affiliations:** Institute of Health & Society, Newcastle University, Baddiley Clark Building, Richardson Road, Newcastle Upon Tyne, NE2 4AA UK

**Keywords:** Physical activity, Biomarkers, Socioeconomic status, UK

## Abstract

**Background:**

Cardiovascular disease (CVD) is the leading cause of global death. Physical activity can help individuals reduce their CVD risk. However, the biological mechanisms explaining the link between physical activity and CVD risk and how they may be mediated by socioeconomic status are not well understood.

**Methods:**

We use cross-sectional data from 2010/2011 of the Understanding Society Survey, UK, to investigate the association between two biomarkers for CVD risk: cholesterol ratio and triglyceride levels and four different measures of physical activity: moderate, mild, self-reported activity rating, and walking 30 min or more a week using multivariate logistic regression. The analysis investigates if this association is mediated by socioeconomic status and difficulty accessing sports facilities.

**Results:**

Results from multivariate regressions show that moderate and self-reported activity rating are significantly associated with cholesterol ratio and triglycerides for both men and women. A weaker association was found for walking 30 min or more a week. No association was found between mild physical activity and the two biomarkers. There is some evidence that socioeconomic status mediates the relationship between the biomarkers and physical activity. A significant association between socioeconomic status variables and the biomarkers was found only for women.

**Conclusions:**

We provide some evidence of the mechanisms explaining the link between CVD risk and physical activity by finding an association with traditional lipid biomarkers. We also find that intensity of physical activity matters. Socioeconomic status especially for women is important which may explain some of the inequalities in CVD risk.

## Key Points

- There is a significant association between lipid biomarkers for CVD risk (cholesterol ratio and triglycerides level) and the frequency and intensity of self-reported and perceived physical activity.
- These data support thenotion that socioeconomic status mediates the relationship between lipid biomarkers and physical activity.
- Low physical activity in lower socioeconomic groups may be contributing to widening health inequalities.
- Promotion of more active transportation may help to encourage physical activity of the required intensity and frequency to improve cardiovascular health.

## Background

The leading cause of global mortality is cardiovascular disease (CVD). There is a well-established link between CVD risk and physical activity [1–3]. The underlying biological mechanisms explaining the link between CVD and physical activity are not fully understood. One previous study [4] investigated the relationship between biomarkers, physical activity, and CVD risk and found that traditional lipids explained approximately 19 % of CVD risk reduction for those who engaged in more than 200 kcal/week of physical activity.

There is currently no evidence if this physiological pathway holds for different types of physical activity. Current physical activity recommendation from the Centre for Disease Control (CDC) [5] suggests that adults participate in at least 2 days of muscle strengthening activities and 150 min of moderate intensity aerobic activity or 75 min of vigorous intensive physical activity each week. If these types of activities have different relationships with lipids associated with increased CVD risk, this may impact on how individuals should engage with physical activity. Additionally, there is no evidence if factors such as socioeconomic status and perceived difficulty in accessing sports facilities attenuate this relationship. This may be a mechanism explaining inequalities in CVD risk.

The aims of this study are to determine if there is an association between two biomarkers for CVD risk: cholesterol ratio and triglyceride levels and four different measures of physical activity: moderate, mild, self-reported activity rating, and walking 30 min or more a week and if this association is mediated by socioeconomic status and difficulty accessing sports facilities using a nationally representative sample of 4823 individuals.

## Methods

### Source Data

We use cross-sectional data from the second round of data collection (2010/2011) of the Understanding Society Survey [6] (*n* = 54,587). Understanding Society is a longitudinal household panel survey of approximately 40,000 household in the UK which began in 2009 [7]. Individual participants are interviewed annually on diverse topics such as health, work, education, income, family, and social life. Further information on the study design and sampling methodology are discussed elsewhere [8]. In the second round of data collection, a representative sub-set of the main sample participated in a nurse-led health assessment (*n* = 15,777) [9]. A total of 13,107 respondents had data on at least one biomarker. For this study, we further limited the sample to respondents that had valid measures for triglycerides, HDL cholesterol, and total cholesterol biomarkers (*n* = 12,867). The final restriction placed on our sample was that participants needed to have valid measures on socioeconomic status, physical activity, and demographic characteristics reducing our sample to *n* = 4823.

Comparing CVD biomarkers between the two groups of the sample population and those who were excluded from the analysis because of missing SES variables generated similar results. The only exemptions were the education variables (where a higher number of people had a less healthy triglyceride level among those with missing data) and access to a car (where a higher number of people had a healthier level of both triglycerides and cholesterol ratio); for both variables, the difference in the number of individuals between groups was less than 5 %. As the nurse assessment sample use for the analysis was chosen to be nationally representative, this suggests that our results should be fairly a representative of the target population as there is less than a 5 % difference between those who reported the SES variables and those that were missing for whatever reason.

Ethical approval was not required for the secondary analysis of this anonymised data source. Respondents provided written consent for their blood to be taken and to be stored for future scientific and genetic analyses [10].

### Outcomes and Key Variables

Biomarkers for CVD risk that were included as key outcome variables were cholesterol ratio [11] and triglyceride levels [12]. Different cholesterol levels were measured from blood serum using enzymatic methods with a Roche module P analyser calibrated to CDC guidelines [10]. Triglycerides were measured from serum blood using an enzymatic method on a Roche P module analyser [10]. Individual total cholesterol and HDL cholesterol level were used to calculate the cholesterol ratio (as $\frac{\mathrm{HDL}\ \mathrm{cholesterol}}{\mathrm{total}\ \mathrm{cholesterol}}$) which was classified as a binary variable equal to 0 if the ratio of HDL to total cholesterol was less than or equal to 3.8 mmol/L (a healthy HDL cholesterol ratio) and equal to 1 if the cholesterol ratio was greater than or equal to 3.9 mmol/L (an unhealthy HDL cholesterol ratio) [13]. Triglycerides were classified as a binary variable where the base category was between 0.3 and 1.9 mmol/L and was equal to 1 if triglycerides were between 2 and 31.9 mmol/L [14].

Three different measures of physical activity were used in the main analysis. Moderate intensity physical activity was defined based upon a positive response to engaging in 29 sports activities that would classify as moderate activity [7]. A binary variable was created that equaled zero if the respondent engaged in moderate activity less than three times a week and was equal to 1 if the respondent engaged in moderate activity three or more times a week. The second measure was a self-assessed sports activity rating where individuals rated on a scale of 1 to 10 how active they were through leisure-based sport. This was classified as a binary variable for high activity which was equal to 0 if respondent scored themselves a 4 or less and was equal to 1 if respondents reported a score of between 5 and 10. The final physical activity variable captured individual walking activity. A binary variable was created that was equal to 0 if respondents walked for 30 min (or less) for four times during the last 4 weeks and was equal to 1 if respondents walked more than 30 min for at least four times in the last 4 weeks [15]. As a validity check on our findings, we used a measure of mild physical activity that should not be significantly associated with reducing CVD risk. Mild intensity physical activity was based upon individuals reporting that they engaged in a sporting activity that would require mild exertion. This was classified as a binary variable that was equal to 0 if respondents engaged in mild activity less than three times a week and was equal to 1 if respondent participated in mild activity three or more times a week.

We controlled for a number of other factors that may confound the relationship between the biomarkers for CVD risk and physical activity participation. The biomarkers used in this analysis, especially triglycerides [10], may have been affected by medications and consumption of food or drink. We therefore controlled for the individual currently taking lipid reducing medication and if they had eaten 30 min before blood was taken. Demographic factors such as age, age squared, marital status, presence of children under the age of 12 in the household, and region [16] were included in the analysis. To determine if the relationship between biomarkers and physical activity were mediated by socioeconomic status and difficulty accessing sports facilities, in some model specifications, socioeconomic status was measured by binary variables for having access to a car or van, owning one’s house or having a mortgage on it, if the individual was employed, highest level of educational attainment achieved, and log of equivalised household income [17]. In addition, in some model specifications, we included a binary variable for if the respondent reports difficulty in accessing sports facilities [14].

### Statistical Analysis

Descriptive analysis was undertaken to gain a better understanding of the prevalence of unhealthy cholesterol ratio and triglyceride levels and to identify physical activity levels and patterning of the confounding variables in the study population. These findings were used to inform the multivariate analysis.

We also performed a number of different multicollinearity tests between the physical activity variables and separately for the socioeconomic status variables. For the physical activity variables, the correlation within different intensities of physical activity was less than 0.40, suggesting that there was no evidence for correlations between the different physical activity measures. Therefore, separate consideration in different models was considered appropriate to describe the behaviours and associations with CVD risk among the sample population. We also tested for multicollinearity between the different socioeconomic status (SES) variables. Correlations between all seven SES variables were very small less than 0.1, the correlation between income and being educated to a degree level was 0.23, and there was a slightly higher correlation between the different educational levels of just above 0.40 which is to be expected as one level of educational attainment is usually correlated with lower levels of educational attainment.

The basic statistical analysis involved multivariate logistic regression models in which the two biomarkers for CVD are a function of one of the four physical activity variables as well as cofounding variables including demographic characteristics, currently taking lipid lower medication, if the respondent has eaten a half hour before blood is taken. To determine if socioeconomic status attenuates the relationship between physical activity and the biomarkers, variables related to socioeconomic status were added to the basic model. Finally, a variable controlling for difficulty in accessing sports facilities was added to the logistic regression. Significant differences were found between physical activity and gender for all types of physical activity except walking (*χ*^2^, *p* = 0.000). Significant differences by gender were found in the outcome variables of cholesterol level and triglyceride levels (*t* test, *p* = 0.000). All analysis was therefore stratified by gender. Survey respondents with missing responses to any of the outcome or explanatory variables required for the analysis were excluded. The analysis was undertaken in Stata v.13 [18].

## Results

Table 1 showed the descriptive analysis of the raw data. Approximately 25 % of men and 21 % of women engaged in at least 30 min of moderate physical activity, three times a week. Approximately 42 % of men and 33 % of women reported being highly active in sports activity. Fifty-seven percent of men and 59 % of women walked at least 30 min a week, and 14 % of men and 18 % of women engaged in mild activity for 30 min or more, three times a week. Approximately 45 % of men and 25 % of women had an unhealthy cholesterol ratio, and 41 % of men and 24 % of women had unhealthy triglyceride levels. The mean age of the sample was 51 years old. The majority of survey respondents had a university education, access to a car, was married, and owned their own home. Approximately 5 % of men and 8 % of women reported difficulty in accessing sports facilities.

**Table 1:** Distribution of outcome variables (cholesterol ratio and triglyceride level), exposure variables (physical activity), and confounding variables

Variable and category	Men	Women
	Mean (SD) obs^e,f,g^	Mean (SD) obs^e,f,g^
Unhealthy cholesterol ratio	0.45 (0.50) 5596	0.25 (0.43) 7091
Unhealthy triglyceride level	0.41 (0.49) 5750	0.24 (0.43) 7139
Taking lipid lowering medication	0.21 (0.41) 4266	0.14 (0.35) 5351
Eaten 30 min before bloods taken	0.09 (0.28) 4266	0.09 (0.29) 5351
Moderate activity^a^	0.25 (0.44) 2941	0.21 (0.41) 3312
Highly active^b^	0.42 (0.50) 4263	0.33 (0.47) 5350
Walk 30 minutes^c^	0.57 (0.50) 3686	0.59 (0.49) 4594
Mild activity^a^	0.14 (0.35) 2858	0.18 (0.38) 3190
Age (years)	51.87 (17.57) 5750	51.31 (16.89) 7139
Married	0.60 (0.49) 5750	0.54 (0.50) 7139
Has children under 12	0.20 (0.40) 5750	0.24 (0.43) 7138
Region (1–16 categories)^d^	1.12 (0.46) 4266	1.13 (0.47) 5351
Home ownership	0.78 (0.41) 5744	0.75 (0.43) 7133
Equivalised household income (£)	18022.07 (14653.98) 5743	16562.56 (13109.78) 7132
Employed	0.59 (0.49) 5750	0.52 (0.50) 7139
University educated	0.39 (0.49) 5050	0.39 (0.49) 6294
Some higher education	0.26 (0.44) 5050	0.18 (0.38) 6294
GCSEs	0.22 (0.41) 5050	0.25 (0.43) 6294
Car ownership	0.93 (0.24) 5008	0.94 (0.25) 5337
Has difficulty accessing PA facilities	0.05 (0.22) 4253	0.08 (0.28) 5335

^a^These variables report the number of respondents engaging in this type of physical activity for at least 30 min, three times a week

^b^High active reports the number of respondents that report 5 or higher for engaging in sport activity (0 to 10 scale)

^c^This variable reports respondents that walk at least 30 min a week

^d^Mean of all regions is shown. Regions were coded 1-England, 2-Scotland, 3-Wales

^e^Base categories for all variables are not shown

^f^Raw data is shown

^g^Values are percentages unless otherwise stated in the footnotes above

Table 2 showed the results of gender-stratified logistic regressions to investigate the relationship between the two biomarkers for CVD risk and moderate physical activity accounting for socioeconomic status. In all models, except for the female triglyceride models adjusted for socioeconomic status and difficulty accessing sports facilities, there is a negative and significant association between moderate physical activity and having an unhealthy cholesterol ratio and triglyceride levels. The size of the association is similar for men and women. This provided some evidence that socioeconomic status attenuates this relationship for cholesterol ratio for men and triglyceride level for women.

**Table 2:** Gender-stratified models of the association between moderate physical activity and biomarkers for CVD risk

	Cholesterol to HDL ratio						Triglycerides					
	Men			Women			Men			Women		
	Base model	SES adjusted model	Full adjusted model	Base model	SES adjusted model	Full adjusted model	Base model	SES adjusted model	Full adjusted model	Base model	SES adjusted model	Full adjusted model
Moderate activity level	0.64*** (−0.63 to −0.27)	0.63*** (−0.67 to −0.26)	0.62*** (−0.68 to −0.28)	0.67*** (−0.62 to −0.17)	0.67*** (−0.66 to −0.14)	0.67*** (−0.66 to −0.14)	0.72*** (−0.51 to −0.16)	0.72*** (−0.52 to −0.12)	0.72*** (−0.53 to −0.13)	0.76** (−0.49 to −0.05)	0.77 (−0.52 to 0.00)	0.77 (−0.52 to 0.00)
Taking lipid lowering medication	0.36*** (−1.28 to −0.77)	0.33*** (−1.40 to −0.83)	0.32*** (−1.42 to −0.84)	0.55*** (−0.93 to −0.25)	0.50*** (−1.14 to −0.24)	0.52*** (−1.11 to −0.22)	1.05 (−0.18 to 0.28)	1.04 (−0.22 to 0.30)	1.03 (−0.23 to 0.30)	1.73*** (0.27 to 0.83)	1.75*** (0.20 to 0.92)	1.73*** (0.18 to 0.91)
Eaten 30 min before bloods taken	0.89 (−0.38 to 0.15)	0.89 (−0.41 to 0.18)	0.90 (−0.41 to 0.19)	0.85 (−0.45 to 0.13)	0.85 (−0.51 to 0.17)	0.85 (−0.50 to 0.17)	1.18 (−0.09 to 0.42)	1.16 (−0.14 to 0.43)	1.15 (−0.14 to 0.43)	1.32** (0.00 to 0.56)	1.38** (0.00 to 0.64)	1.38** (0.00 to 0.64)
Age	1.18*** (0.14 to 0.20)	1.17*** (0.12 to 0.20)	1.17*** (0.12 to 0.20)	1.07*** (0.03 to 0.10)	1.09*** (0.04 to 0.14)	1.09*** (0.04 to 0.14)	1.15*** (0.11 to 0.17)	1.14*** (0.09 to 0.17)	1.14*** (0.09 to 0.17)	1.08*** (0.05 to 0.12)	1.18*** (0.11 to 0.22)	1.18*** (0.11 to 0.22)
Married	0.88 (−0.31 to 0.06)	0.82 (−0.42 to 0.01)	0.81 (−0.42 to 0.00)	0.95 (−0.24 to 0.13)	1.10 (−0.13 to 0.32)	1.10 (−0.13 to 0.31)	0.88 (−0.31 to 0.05)	0.82 (−0.40 to 0.01)	0.82 (−0.41 to 0.00)	1.06 (−0.13 to 0.25)	1.07 (−0.16 to 0.29)	1.07 (−0.16 to 0.29)
Has children under 12	1.17 (−0.05 to 0.36)	1.09 (−0.15 to 0.33)	1.09 (−0.15 to 0.33)	1.05 (−0.17 to 0.27)	0.97 (−0.30 to 0.24)	0.95 (−0.33 to 0.22)	1.07 (−0.13 to 0.27)	1.01 (−0.22 to 0.24)	1.02 (−0.21 to 0.25)	1.06 (−0.17 to 0.29)	1.07 (−0.22 to 0.35)	1.05 (−0.24 to 0.34)
Home ownership		1.13 (−0.13 to 0.38)	1.15 (−0.12 to 0.39)		0.74** (−0.59 to −0.01)	0.75 (−0.58 to 0.01)		1.05 (−0.20 to 0.29)	1.05 (−0.20 to 0.30)		0.62*** (−0.78 to −0.18)	0.61*** (−0.79 to −0.19)
Equivalised household income		0.87 (−0.30 to 0.01)	0.87 (−0.30 to 0.01)		0.84** (−0.34 to −0.02)	0.84** (−0.33 to −0.02)		0.92 (−0.22 to 0.06)	0.92 (−0.22 to 0.06)		0.85** (−0.32 to −0.01)	0.85** (−0.32 to −0.01)
Employed		1.17 (−0.12 to 0.44)	1.17 (−0.12 to 0.43)		1.01 (−0.25 to 0.27)	1.02 (−0.24 to 0.28)		1.20 (−0.09 to 0.45)	1.19 (−0.09 to 0.44)		0.95 (−0.32 to 0.22)	0.95 (−0.32 to 0.22)
College educated		0.78 (−0.62 to 0.12)	0.78 (−0.62 to 0.12)		0.62** (−0.89 to −0.07)	0.63** (−0.88 to −0.06)		1.08 (−0.28 to 0.43)	1.06 (−0.30 to 0.41)		0.77 (−0.68 to 0.15)	0.79 (−0.65 to 0.19)
Some higher education		0.90 (−0.49 to 0.28)	0.91 (−0.48 to 0.29)		0.55*** (−1.06 to −0.15)	0.56** (−1.04 to −0.12)		1.21 (−0.18 to 0.56)	1.19 (−0.19 to 0.55)		0.70 (−0.82 to 0.11)	0.73 (−0.78 to 0.15)
High school qualification		1.11 (−0.29 to 0.50)	1.11 (−0.29 to 0.50)		0.67 (−0.83 to 0.03)	0.69 (−0.80 to 0.06)		1.29 (−0.13 to 0.64)	1.28 (−0.14 to 0.63)		0.82 (−0.64 to 0.23)	0.85 (−0.61 to 0.27)
Car ownership		1.15 (−0.25 to 0.53)	1.16 (−0.24 to 0.54)		0.77 (−0.72 to 0.18)	0.79 (−0.69 to 0.21)		1.07 (−0.31 to 0.44)	1.05 (−0.33 to 0.43)		0.95 (−0.54 to 0.43)	1.00 (−0.49 to 0.49)
Has difficulty accessing PA facilities			1.23 (−0.41 to 0.82)			1.74** (0.08 to 1.02)			0.76 (−0.88 to 0.34)			1.38 (−0.19 to 0.83)
Observations	2860	2280	2278	3254	2461	2459	2939	2330	2328	3309	2498	2496

Confidence intervals are in parentheses. Regional dummy variables are included in the adjusted models but not shown

***p < 0.01, **p < 0.05

For both men and women, age was associated with an increased likelihood of having an unhealthy cholesterol ratio and triglyceride levels. For both genders, taking lipid lowering medication was associated with an increased likelihood of having a healthy cholesterol level. For women only, taking lipid lower medication was significantly associated with having unhealthy triglyceride levels. This may be picking up women, who were on lipid-lowering medication because they have been recently diagnosed with a high triglyceride level. Eating 30 min before the interview was positively and significantly associated with having an unhealthy triglyceride level. For women only, a number of socioeconomic factors were significantly associated with cholesterol ratio and triglyceride levels. Being a home owner compared to renting was negatively associated with unhealthy cholesterol ratio in the model adjusted for socioeconomic status and with triglyceride levels in the model adjusted for socioeconomic status and access. Household income was negatively and significantly associated with unhealthy cholesterol ratio and triglyceride levels in all adjusted models. Being educated to the college degree level or having some higher education was negatively and significantly associated with having an unhealthy cholesterol ratio in all adjusted models. Reporting difficulty in accessing sports facilities was positively and significantly associated with an unhealthy cholesterol ratio in the model adjusted for access.

Table 3 displayed the results of gender-stratified regressions to investigate the association between self-reported activity rating and the two biomarkers for CVD risk and socioeconomic status. Reporting being highly active through sport was negatively associated with increased likelihood of having an unhealthy cholesterol ratio and triglyceride levels. This provided evidence that the relationship between activity and cholesterol ratio may be mediated by socioeconomic status for both genders. The relationship between triglyceride levels and activity was only mediated by socioeconomic status for men.

**Table 3:** Gender-stratified models of the association between self-reported activity rating and biomarkers for CVD risk

	Cholesterol to HDL ratio						Triglycerides					
	Men			Women			Men			Women		
	Base model	SES adjusted model	Full adjusted model	Base model	SES adjusted model	Full adjusted model	Base model	SES adjusted model	Full adjusted model	Base model	SES adjusted model	Full adjusted model
High activity rating	0.69*** (−0.51 to −0.24)	0.66*** (−0.58 to −0.26)	0.66*** (−0.57 to −0.26)	0.50*** (−0.84 to −0.54)	0.54*** (−0.80 to −0.44)	0.54*** (−0.80 to −0.44)	0.73*** (−0.44 to −0.18)	0.70*** (−0.51 to −0.21)	0.70*** (−0.51 to −0.21)	0.57*** (−0.71 to −0.41)	0.56*** (−0.77 to −0.40)	0.56*** (−0.76 to −0.40)
Taking lipid lowering medication	0.34*** (−1.27 to −0.90)	0.30*** (−1.43 to −1.00)	0.29*** (−1.44 to −1.01)	0.50*** (−0.90 to −0.47)	0.52*** (−0.94 to −0.36)	0.53*** (−0.93 to −0.35)	0.94 (−0.23 to 0.11)	0.91 (−0.29 to 0.10)	0.91 (−0.30 to 0.10)	1.49*** (0.22 to 0.58)	1.46*** (0.13 to 0.63)	1.46*** (0.13 to 0.63)
Eaten 30 min before bloods taken	0.88 (−0.36 to 0.10)	0.88 (−0.39 to 0.14)	0.88 (−0.39 to 0.14)	0.95 (−0.27 to 0.17)	0.90 (−0.38 to 0.17)	0.90 (−0.38 to 0.17)	1.13 (−0.10 to 0.34)	1.09 (−0.17 to 0.34)	1.09 (−0.17 to 0.34)	1.26** (0.01 to 0.45)	1.41** (0.08 to 0.61)	1.41** (0.08 to 0.61)
Age	1.16*** (0.13 to 0.17)	1.17*** (0.12 to 0.19)	1.17*** (0.12 to 0.19)	1.11*** (0.08 to 0.13)	1.13*** (0.08 to 0.17)	1.13** (0.08 to 0.17)*	1.13*** (0.10 to 0.15)	1.13*** (0.09 to 0.15)	1.13*** (0.09 to 0.15)	1.12*** (0.08 to 0.14)	1.17*** (0.11 to 0.20)	1.17*** (0.11 to 0.20)
Married	0.94 (−0.21 to 0.09)	0.86 (−0.33 to 0.03)	0.86 (−0.33 to 0.02)	0.95 (−0.18 to 0.08)	1.07 (−0.11 to 0.25)	1.08 (−0.10 to 0.25)	0.93 (−0.22 to 0.07)	0.89 (−0.29 to 0.05)	0.89 (−0.29 to 0.05)	1.02 (−0.11 to 0.16)	1.03 (−0.15 to 0.20)	1.03 (−0.14 to 0.21)
Has children under 12	1.21** (0.01 to 0.37)	1.14 (−0.08 to 0.35)	1.14 (−0.08 to 0.35)	0.92 (−0.27 to 0.10)	0.84 (−0.41 to 0.06)	0.83 (−0.42 to 0.05)	1.10 (−0.08 to 0.27)	1.05 (−0.16 to 0.26)	1.05 (−0.16 to 0.26)	0.94 (−0.25 to 0.14)	0.97 (−0.28 to 0.22)	0.96 (−0.30 to 0.21)
House ownership		1.09 (−0.12 to 0.29)	1.10 (−0.12 to 0.30)		0.68*** (−0.61 to −0.17)	0.68*** (−0.60 to −0.16)		1.00 (−0.20 to 0.20)	1.01 (−0.20 to 0.21)		0.66*** (−0.64 to −0.18)	0.66*** (−0.64 to −0.19)
Income		0.92 (−0.20 to 0.04)	0.92 (−0.20 to 0.04)		0.88** (−0.25 to −0.00)	0.88 (−0.25 to 0.00)		1.01 (−0.11 to 0.12)	1.01 (−0.11 to 0.12)		0.87** (−0.26 to −0.02)	0.87** (−0.26 to −0.01)
In employment		1.11 (−0.12 to 0.32)	1.11 (−0.12 to 0.33)		0.80** (−0.43 to −0.02)	0.80** (−0.42 to −0.02)		1.08 (−0.14 to 0.29)	1.08 (−0.14 to 0.29)		0.84 (−0.38 to 0.03)	0.85 (−0.37 to 0.04)
Degree level qualification or higher		0.82 (−0.46 to 0.06)	0.83 (−0.45 to 0.07)		0.70** (−0.64 to −0.09)	0.70** (−0.63 to −0.08)		0.91 (−0.35 to 0.16)	0.91 (−0.34 to 0.16)		0.87 (−0.41 to 0.14)	0.89 (−0.39 to 0.16)
A-level qualification or equivalent		0.88 (−0.41 to 0.14)	0.89 (−0.39 to 0.16)		0.70** (−0.67 to −0.05)	0.71** (−0.66 to −0.03)		1.08 (−0.18 to 0.34)	1.09 (−0.18 to 0.35)		0.79 (−0.55 to 0.08)	0.81 (−0.53 to 0.11)
GCSE qualification or equivalent		1.13 (−0.16 to 0.41)	1.14 (−0.15 to 0.42)		0.82 (−0.49 to 0.08)	0.82 (−0.48 to 0.09)		1.14 (−0.14 − 0.41)	1.15 (−0.14 − 0.41)		0.95 (−0.33 to 0.23)	0.97 (−0.31 to 0.26)
Car ownership		1.33 (−0.04 to 0.60)	1.36 (−0.02 to 0.63)		0.80 (−0.55 to 0.11)	0.80 (−0.55 to 0.11)		1.12 (−0.19 to 0.42)	1.12 (−0.20 to 0.42)		0.88 (−0.46 to 0.21)	0.90 (−0.44 to 0.24)
Has difficulty accessing PA facilities			1.30 (−0.15 to 0.67)			1.15 (−0.20 to 0.49)			0.99 (−0.40 to 0.38)			1.27 (−0.11 to 0.58)
Observations	4144	3172	3168	5258	3511	3505	4261	3248	3244	5346	3564	3557

Confidence intervals are in parentheses. Regional dummy variables are included in the adjusted models but not shown

***p < 0.01, **p < 0.05

Similar to the results found in Table 2, age was associated with a higher likelihood of having an unhealthy cholesterol level and triglyceride levels. In Table 3, the probability was similar for both genders. Socioeconomic factors were only significantly associated with cholesterol and triglyceride level risk for women. The same associations as found in Table 2 are also found in Table 3. The exception is in Table 3, being employed compared to not being in the labour market was negatively and significantly associated with having an unhealthy cholesterol ratio. And access to sports facility was no longer significantly associated with cholesterol ratio for women.

Table 4 showed the results from the gender-stratified models of the relationship between CVD risk, walking, and socioeconomic status. Walking 30 min a week or more was negatively associated with having an unhealthy cholesterol ratio for men in the unadjusted model. This association held for all models for women. There is some evidence that this relationship was mediated by socioeconomic status for both genders. For men in all models, there was a negative and significant association between walking 30 min a week or more and triglyceride levels. There was no significant association with walking and triglyceride levels for women. The significance of the relationship between the variables related to socioeconomic status and CVD risk was similar to those found in Table 2 and Table 3.

**Table 4:** Gender stratified models of the association between walking 30 min or more a week and biomarkers for CVD risk

	Cholesterol to HDL ratio						Triglycerides					
	Men			Women			Men			Women		
	Base model	SES adjusted model	Full adjusted model	Base model	SES adjusted model	Full adjusted model	Base model	SES adjusted model	Full adjusted model	Base model	SES adjusted model	Full adjusted model
Walking	0.85** (−0.30 to −0.02)	0.89 (−0.28 to 0.04)	0.88 (−0.29 to 0.04)	0.79*** (−0.37 to −0.09)	0.82** (−0.38 to −0.03)	0.82** (−0.38 to −0.03)	0.81*** (−0.34 to −0.07)	0.82** (−0.35 to −0.04)	0.82** (−0.36 to −0.05)	0.90 (−0.24 to 0.04)	0.89 (−0.29 to 0.06)	0.89 (−0.29 to 0.07)
Taking lipid lowering medication	0.32*** (−1.33 to −0.92)	0.30*** (−1.45 to −0.97)	0.29*** (−1.46 to −0.98)	0.46*** (−1.03 to −0.52)	0.46*** (−1.11 to −0.43)	0.47*** (−1.10 to −0.41)	0.95 (−0.24 to 0.14)	0.95 (−0.27 to 0.17)	0.95 (−0.27 to 0.16)	1.56*** (0.24 to 0.65)	1.40** (0.05 to 0.63)	1.40** (0.05 to 0.62)
Eaten 30 min before bloods taken	0.82 (−0.45 to 0.05)	0.85 (−0.45 to 0.12)	0.85 (−0.45 to 0.12)	0.90 (−0.35 to 0.14)	0.88 (−0.42 to 0.17)	0.88 (−0.43 to 0.17)	1.04 (−0.20 to 0.28)	1.01 (−0.26 to 0.29)	1.02 (−0.26 to 0.29)	1.25 (−0.01 to 0.46)	1.39** (0.05 to 0.61)	1.39** (0.05 to 0.61)
Age	1.17*** (0.13 to 0.18)	1.16*** (0.11 to 0.19)	1.16*** (0.11 to 0.19)	1.12*** (0.08 to 0.14)	1.13*** (0.08 to 0.17)	1.13*** (0.08 to 0.17)	1.14*** (0.11 to 0.16)	1.14*** (0.10 to 0.16)	1.14*** (0.09 to 0.16)	1.13*** (0.09 to 0.15)	1.18*** (0.12 to 0.22)	1.19*** (0.12 to 0.22)
Married	0.89 (−0.28 to 0.04)	0.83** (−0.38 to −0.00)	0.82** (−0.39 to −0.00)	0.90 (−0.25 to 0.04)	1.06 (−0.14 to 0.25)	1.06 (−0.14 to 0.25)	0.89 (−0.27 to 0.04)	0.86 (−0.33 to 0.03)	0.86 (−0.33 to 0.03)	1.02 (−0.13 to 0.17)	1.05 (−0.15 to 0.24)	1.05 (−0.14 to 0.25)
Has children under 12	1.20 (−0.01 to 0.37)	1.11 (−0.13 to 0.33)	1.10 (−0.13 to 0.33)	0.99 (−0.20 to 0.19)	0.88 (−0.38 to 0.12)	0.87 (−0.39 to 0.11)	1.09 (−0.10 to 0.28)	1.01 (−0.21 to 0.23)	1.01 (−0.21 to 0.23)	0.97 (−0.23 to 0.18)	0.96 (−0.31 to 0.23)	0.94 (−0.33 to 0.21)
House ownership		1.14 (−0.10 to 0.36)	1.15 (−0.09 to 0.37)		0.64*** (−0.70 to −0.21)	0.64*** (−0.70 to −0.21)		1.09 (−0.14 to 0.31)	1.09 (−0.13 to 0.31)		0.63*** (−0.71 to −0.21)	0.63*** (0.71 to −0.21)
Income		0.90 (−0.22 to 0.02)	0.91 (−0.22 to 0.02)		0.88 (−0.25 to 0.00)	0.89 (−0.25 to 0.01)		0.99 (−0.12 to 0.11)	0.99 (−0.13 to 0.11)		0.86** (−0.28 to −0.02)	0.87** (−0.27 to −0.01)
In employment		1.22 (−0.04 to 0.44)	1.22 (−0.04 to 0.44)		0.82 (−0.41 to 0.03)	0.82 (−0.42 to 0.03)		1.16 (−0.09 to 0.38)	1.16 (−0.09 to 0.38)		0.81 (−0.43 to 0.01)	0.81 (−0.43 to 0.01)
College Educated		0.84 (−0.47 to 0.12)	0.85 (−0.46 to 0.14)		0.65*** (−0.73 to −0.12)	0.66*** (−0.72 to −0.11)		0.89 (−0.40 to 0.17)	0.89 (−0.40 to 0.17)		0.76* (−0.57 to 0.03)	0.78 (0.55 to 0.06)
Some higher education		0.89 (−0.43 to 0.19)	0.90 (−0.42 to 0.21)		0.63** (−0.81 to −0.11)	0.64** (−0.80 to −0.09)		1.03 (−0.27 to 0.33)	1.03 (−0.27 to 0.33)		0.69** (−0.72 to −0.02)	0.72 (−0.69 to 0.02)
High school qualification		1.26 (−0.09 to 0.56)	1.28 (−0.08 to 0.58)		0.78 (−0.56 to 0.08)	0.79 (−0.55 to 0.09)		1.14 (−0.18 to 0.44)	1.14 (−0.18 to 0.45)		0.85 (−0.47 to 0.16)	0.88 (−0.45 to 0.19)
Car ownership		1.17 (−0.18 to 0.50)	1.20 (−0.16 to 0.53)		0.71 (−0.69 to 0.02)	0.71 (−0.69 to 0.02)		1.06 (−0.27 to 0.39)	1.06 (−0.28 to 0.38)		0.72 (−0.69 to 0.03)	0.74 (−0.66 to 0.06)
Has difficulty accessing PA facilities			1.41 (−0.14 to 0.83)			1.22 (−0.21 to 0.61)			0.96 (−0.51 to 0.43)			1.53** (0.03 to 0.82)
Observations	3582	2743	2739	4506	3051	3047	3684	2812	2808	4590	3102	3097

Confidence intervals are in parentheses. Regional dummy variables are included in the adjusted models but not shown

***p < 0.01, **p < 0.05

As can be seen in Table 5, there was no significant association between mild activity and the biomarkers for CVD risk for either gender.

**Table 5:** Gender-stratified models of the association between mild physical activity and biomarkers for CVD risk

	Cholesterol to HDL ratio						Triglycerides					
	Men			Women			Men			Women		
	Base model	SES adjusted model	Full adjusted model	Base model	SES adjusted model	Full adjusted model	Base model	SES adjusted model	Full adjusted model	Base model	SES adjusted model	Full adjusted model
Mild activity level	1.06 (−0.18 to 0.29)	1.12 (−0.16 to 0.38)	1.11 (−0.17 to 0.37)	0.93 (−0.30 to 0.15)	0.92 (−0.35 to 0.19)	0.93 (−0.34 to 0.20)	0.87 (−0.37 − 0.08)	0.86 (−0.41 to 0.11)	0.85 (−0.42 to 0.10)	0.99 (−0.23 to 0.22)	0.91 (−0.37 to 0.18)	0.91 (−0.36 to 0.18)
Taking lipid-lowering medication	0.36*** (−1.28 to −0.78)	0.32*** (−1.42 to −0.85)	0.32*** (−1.43 to −0.86)	0.50*** (−1.04 to −0.34)	0.40*** (−1.36 to −0.45)	0.41*** (−1.35 to −0.44)	0.99 (−0.24 to 0.22)	0.92 (−0.34 to 0.18)	0.92 (−0.34 to 0.17)	1.32 (−0.01 to 0.57)	1.25 (−0.14 to 0.59)	1.26 (−0.14 to 0.59)
Eaten 30 min before bloods taken	0.76 (−0.55 to 0.00)	0.76 (−0.59 to 0.04)	0.76 (−0.58 to 0.04)	0.99 (−0.31 to 0.28)	0.89 (−0.47 to 0.23)	0.89 (−0.47 to 0.23)	1.16 (−0.12 to 0.41)	1.11 (−0.19 to 0.40)	1.11 (−0.19 to 0.40)	1.38** (0.04 to 0.61)	1.53** (0.10 to 0.75)	1.52** (0.10 to 0.74)
Age	1.17*** (0.13 to 0.19)	1.18*** (0.13 to 0.21)	1.18*** (0.13 to 0.21)	1.11*** (0.06 to 0.14)	1.13*** (0.07 to 0.18)	1.13*** (0.07 to 0.18)	1.14*** (0.11 − 0.16)	1.15*** (0.10 to 0.18)	1.15*** (0.10 − 0.18)	1.11*** (0.06 to 0.14)	1.20*** (0.12 to 0.24)	1.20*** (0.13 to 0.25)
Married	0.86 (−0.33 to 0.03)	0.81 (−0.42 to 0.01)	0.81 (−0.42 to 0.01)	0.86 (−0.33 to 0.03)	1.00 (−0.22 to 0.23)	1.01 (−0.21 to 0.24)	0.81** (−0.38 − −0.03)	0.82 (−0.40 to 0.00)	0.82 (−0.40 to 0.01)	1.05 (−0.14 to 0.23)	1.00 (−0.22 to 0.23)	1.01 (−0.22 to 0.24)
Has children under 12	1.28** (0.04 to 0.46)	1.19 (−0.07 to 0.42)	1.18 (−0.08 to 0.42)	1.08 (−0.16 to 0.31)	1.00 (−0.29 to 0.29)	0.98 (−0.31 to 0.27)	1.21 (−0.02 − 0.40)	1.11 (−0.14 to 0.34)	1.11 (−0.14 to 0.34)	0.96 (−0.28 − 0.21)	0.98 (−0.33 to 0.28)	0.96 (−0.35 to 0.26)
Home ownership		0.95 (−0.31 to 0.21)	0.95 (−0.31 to 0.21)		0.65*** (−0.72 to −0.14)	0.66*** (−0.70 to −0.12)		0.90 (−0.35 to 0.15)	0.90 (−0.36 to 0.15)		0.65*** (−0.73 to −0.14)	0.65*** (−0.73 to −0.14)
Equivalised household income		0.90 (−0.24 to 0.03)	0.90 (−0.25 to 0.03)		0.87 (−0.30 to 0.02)	0.87 (−0.30 to 0.02)		0.96 (−0.17 to 0.09)	0.96 (−0.17 to 0.09)		0.87 (−0.30 to 0.01)	0.87 (−0.30 to 0.01)
Employed		1.12 (−0.16 to 0.38)	1.12 (−0.16 to 0.39)		0.93 (−0.34 to 0.19)	0.94 (−0.33 to 0.20)		1.07 (−0.20 to 0.32)	1.07 (−0.20 to 0.33)		0.89 (−0.38 to 0.15)	0.89 (−0.38 to 0.15)
College educated		0.92 (−0.45 to 0.29)	0.94 (−0.44 to 0.31)		0.56*** (−0.98 to −0.18)	0.56*** (−0.98 to −0.17)		0.96 (−0.40 to 0.32)	0.98 (−0.38 to 0.34)		0.78 (−0.66 to 0.15)	0.80 (−0.64 to 0.18)
Some higher education		1.24 (−0.17 to 0.61)	1.27 (−0.15 to 0.63)		0.48*** (−1.19 to −0.29)	0.48*** (−1.18 to −0.28)		1.29 (−0.12 to 0.63)	1.31 (−0.11 to 0.64)		0.76 (−0.72 to 0.18)	0.78 (−0.70 to 0.21)
High school qualification		1.44 (−0.04 to 0.76)	1.47 (−0.02 to 0.79)		0.65** (−0.85 to −0.02)	0.66 (−0.84 to 0.01)		1.23 (−0.17 to 0.59)	1.25 (−0.16 to 0.61)		0.92 (−0.51 to 0.34)	0.94 (−0.48 to 0.37)
Car ownership		1.24 (−0.17 to 0.60)	1.26 (−0.16 to 0.62)		0.64** (−0.89 to −0.01)	0.64 (−0.88 to 0.00)		1.07 (−0.31 to 0.44)	1.06 (−0.32 to 0.44)		0.68 (−0.84 to 0.06)	0.69 (−0.83 to 0.08)
Has difficulty accessing PA facilities			1.24 (−0.38 to 0.81)			1.47 (−0.13 to 0.90)			0.99 (−0.58 to 0.57)			1.52 (−0.10 to 0.93)
Observations	2771	2193	2191	3134	2376	2374	2856	2252	2250	3189	2413	2411

Confidence intervals are in parentheses. Regional dummy variables are included in the adjusted models but not shown

***p < 0.01, **p < 0.05

## Discussion

In a population-based cross-sectional sample of adults, we find a significant association between two biomarkers for CVD risk (cholesterol ratio and triglycerides level) and frequency of self-reported moderate physical activity and self-reported perceived level of weekly sports-based activity. A weaker association was found for walking 30 min or more per week. These results are consistent with a large body of literature finding a negative association between physical activity and CVD risk [1, 2, 5, 19]. The findings show that intensity of activity is important [20, 21]. Mild activity even if performed as frequently as moderate activity is not significantly associated with a reduced probability of having unhealthy cholesterol ratio or triglyceride levels. Our results also corroborate findings [4] that traditional lipid biomarker may mediate the relationship between CVD risk and physical activity and provide additional evidence on the mechanisms that may explain the link between CVD risk and physical activity.

Socioeconomic status is likely to be associated with CVD risk through a number of complex pathways that include economic, social, environmental, and biological pathways [22]. We find some evidence that this relationship between our biomarkers for CVD risk and physical activity is mediated by socioeconomic status. Those in lower socioeconomic neighbourhoods may have less opportunities for physical activity [15, 23] leading to widening health inequalities. Other lifestyle and health-related behaviours such as individual diet may be mediated through socioeconomic status and thereby may have affected our outcome variables. There may also be the cumulative negative effect of socioeconomic status on our biomarkers for CVD risk which is being picked up in the analysis [24]. Variables related to socioeconomic status were associated with our biomarkers for CVD risk for women only. The negative associations of socioeconomic status with health are larger for women than for men [25]. It is possible that men in lower socioeconomic status may be employed in manual professionals participating in more physical activity, which could attenuate some of the negative impacts of lower socioeconomic status on the biomarkers for CVD risk.

## Strengths and Limitations

This study used a cross-section of data from the Understanding Society Survey which is a nationally representative dataset providing a good level of generalisability to the UK population. Self-reported measures of physical activity which are easy and cheap to collect continue to be widely used in research [26]. This study showed that these measures are on average significantly associated with objective measures of CVD risk providing some support of the validity of these measures for future research especially in large scale studies where it may not be practical or cost-effective to use objective methods for the measurement of individual physical activity such as accelerometers.

A key limitation of this study is that only 1 year of data with the required variables for this analysis is available limiting our ability to estimate a causal relationship. Longitudinal data would allow us to better understand how physical activity and the lipid biomarkers impact on CVD risk and for example account for changes in medication due to a recent diagnosis of high cholesterol levels.

More detailed information on type, intensity, and duration of physical activity would also be useful for better understanding the relationship between physical activity and lipid biomarkers. There is also a considerable number of missing responses when taking into account all of the requirements required for the statistical model. This may have some impact on the generalisability of the results.

## Conclusions

This study supports the development of general lifestyle interventions and those targeted at women from lower socioeconomic backgrounds to promote moderate physical activity and incorporate physical activity into individual’s daily routine. Local policy to promote active transportation and public transportation use may be one mechanism to promote physical activity of the required intensity [27].
